# [^68^Ga]Ga-tilmanocept PET/CT lymphoscintigraphy for sentinel lymph node detection in early-stage oral cavity carcinoma

**DOI:** 10.1007/s00259-020-05060-x

**Published:** 2020-11-13

**Authors:** Rutger Mahieu, Gerard C. Krijger, F.F. Tessa Ververs, Remmert de Roos, Remco de Bree, Bart de Keizer

**Affiliations:** 1 Department of Head and Neck Surgical Oncology, University Medical Center Utrecht, Utrecht, the Netherlands; 2 Department of Radiology and Nuclear Medicine, University Medical Center Utrecht, Heidelberglaan 100, 3584 CX Utrecht, the Netherlands; 3 Department of Pharmacy, University Medical Center Utrecht, Utrecht, the Netherlands

**Figure 1:**
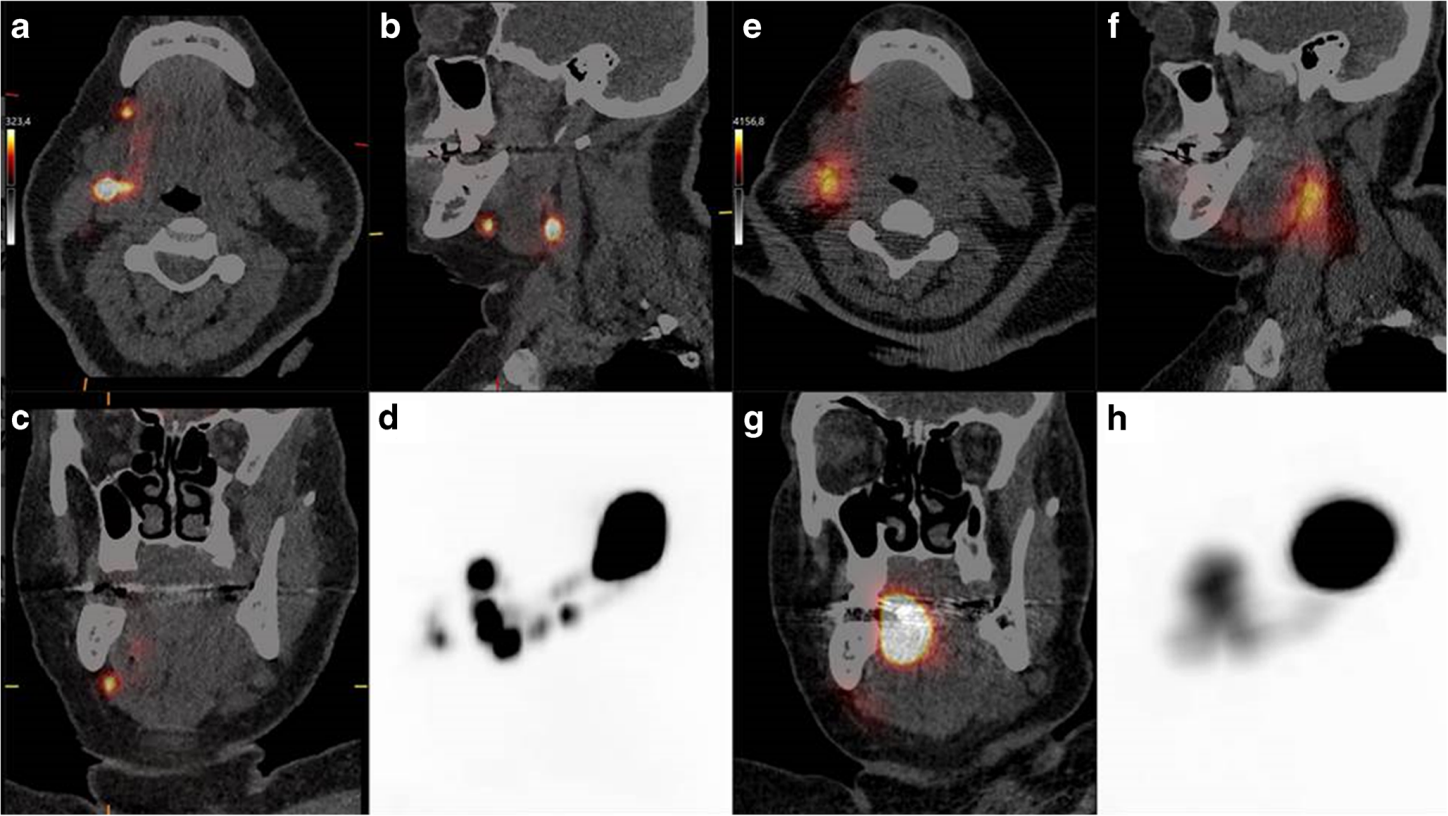
P[^68^Ga]Ga-tilmanocept PET/CT vs. [^99m^Tc]Tc-tilmanocept PET/CT.

The sentinel lymph node (SLN) procedure is routinely performed for nodal staging in several malignancies, including early-stage oral cancer. In oral cancer, the SLN imaging procedure usually consists of peritumoral injections with a [^99m^Tc]Tc-labelled radiotracer followed by dynamic and planar lymphoscintigraphy and SPECT/CT [1, 2]. A frequently discussed limitation of this procedure in oral cancer arises in situations where SLNs are located in close vicinity of the radiotracer injection site. Due to the limited resolution of conventional scintigraphy and SPECT/CT, injection site activity can hide adjacent SLNs and hamper discrimination between injection site and SLNs (shine-through phenomenon), potentially resulting in false-negative SLN procedure outcomes [3, 4]. PET/CT lymphoscintigraphy may offer a solution, as it provides superior spatial resolution compared with conventional scintigraphy and SPECT/CT [4, 5]. Here, we present the first within-patient comparison between PET/CT lymphoscintigraphy using [^68^Ga]Ga-tilmanocept (*10 MBq; 15 min post-injection*) and SPECT/CT with [^99m^Tc]Tc-tilmanocept (*74 MBq; 2 h post-injection*) in a cT1N0 tongue cancer patient, both acquired on the day before surgery. Maximum intensity projection images (MIP) of PET (D) demonstrate its superior resolution compared with SPECT (H). Furthermore, two separate lymph vessels can be identified on PET/CT lymphoscintigraphy (D), which are not visualized on SPECT/CT (H). Also note that the activity in a SLN in level Ib on the right site is better visible on axial (A), sagittal (B), and coronal (C) PET/CT lymphoscintigraphic images compared with corresponding SPECT/CT reconstructions (E,F,G). Surgically, five SLNs were localized and harvested (level Ib, 3x level IIa and level III), using a conventional gammaprobe. Histopathological assessment showed metastasis in one SLN located in level IIa. Complementary neck dissection of level I-IV showed no additional lymphatic metastasis (Figure 1).
